# Butyrate modifies epigenetic and immune pathways in peripheral mononuclear cells from children with neurodevelopmental disorders associated with chromatin dysregulation

**DOI:** 10.1016/j.neurot.2025.e00792

**Published:** 2025-11-19

**Authors:** Jessica P. Hayes, Velda X. Han, Brooke A. Keating, Hiroya Nishida, Erica Tsang, Xianzhong Lau, Ruwani Dissanayake, Nader Aryamanesh, Wendy Gold, Melanie Wong, Carolyn Ellaway, Brian S. Gloss, Shekeeb S. Mohammad, Markus J. Hofer, Peter Valtchev, Shrujna Patel, Russell C. Dale

**Affiliations:** a Kids Neuroscience Centre, The Children's Hospital at Westmead, Faculty of Medicine and Health, University of Sydney, NSW, Australia; b Khoo Teck Puat-National University Children's Medical Institute, National University Health System, Singapore; c Department of Paediatrics, Yong Loo Lin School of Medicine, National University of Singapore, Singapore; d Australian Genome Research Facility Ltd, Melbourne, VIC, Australia; e Australian Genome Research Facility Ltd, Westmead, NSW, Australia; f Bioinformatics Group, Children's Medical Research Institute, University of Sydney, Westmead, NSW, Australia; g The Children's Hospital at Westmead, Department of Allergy and Immunology, Australia; h The Children's Hospital at Westmead, Genetic Metabolic Disorders Service, Australia; i Westmead Research Hub, Westmead Institute for Medical Research, Westmead, NSW, Australia; j The Children's Hospital at Westmead Clinical School, Faculty of Medicine and Health, University of Sydney, Sydney, NSW, Australia; k School of Life and Environmental Sciences, Faculty of Science, The University of Sydney, Sydney, NSW, Australia; l Charles Perkins Centre, The University of Sydney, Sydney, NSW, Australia; m The University of Sydney, School of Medical Sciences and Discipline of Child and Adolescent Health, Faculty of Medicine and Health, Sydney, NSW, Australia; n School of Chemical and Biomolecular Engineering, Faculty of Engineering, The University of Sydney, Sydney, NSW, Australia

**Keywords:** Neurodevelopmental disorders, Neuroregression, Epigenetics, Chromatin, Histone deacetylase inhibition, Butyrate

## Abstract

Pathogenic DNA variants in chromatin-related genes constitute an important minority of neurodevelopmental disorders (NDDs). Epigenetic mechanisms, including chromatin regulation driven by genetic or environmental factors, are increasingly recognised as key contributors to pathogenesis of diverse NDDs. We hypothesise that therapeutic strategies targeting chromatin dysregulation, such as histone deacetylase inhibition with butyrate, may be a potential disease modifying therapy for NDDs. We first performed peripheral blood bulk RNA sequencing (RNA-seq) to explore baseline gene regulation in children with chromatin-related NDDs (Kabuki syndrome (*KMT2D,* n ​= ​4), CHARGE syndrome (*CHD7,* n ​= ​2), and Rett syndrome (*MECP2,* n ​= ​5), and children with NDDs but without a monogenic diagnosis (non-monogenic, n ​= ​8), compared with sex-matched healthy controls (total n ​= ​21). Next, to explore the effects of butyrate, single-cell RNA sequencing (scRNA-seq) was performed on 101,539 peripheral immune cells from four selected patients (one per condition) and two controls, before and after butyrate treatment. At baseline, dysregulation of ribosomal and immune pathways was seen in all four NDD cohorts (*KMT2D, CHD7, MECP2*, non-monogenic) compared to controls. Butyrate largely reversed these pathways, normalising ribosomal and immune pathways in patient and control cells. Butyrate induced up-regulation of ribosome, GTPase, cytoskeletal, mitochondrial pathways, and down-regulation of epigenetic and immune pathways. In conclusion, we identified a common ribosomal-immune RNA signature in chromatin-related NDDs, and a similar signature in non-monogenic NDDs. We showed that butyrate modulates epigenetic and immune gene networks in monogenic and non-monogenic NDDs, positioning butyrate as a promising therapeutic modulator across diverse NDDs.

## Introduction

*De novo* pathogenic DNA variants in chromatin-related genes, such as those causing Kabuki, CHARGE, and Rett syndromes, constitute an important minority of neurodevelopmental disorders (NDDs) [1]. However, NDDs in most children are not due to rare pathogenic DNA variants but are instead thought to arise from interactions between common genetic variations and environmental factors, associated with epigenetic and sometimes immune dysregulation [2]. Environmental exposures are proposed to leave epigenetic marks on the genome, influencing the expression of genes involved in neurodevelopment [3,4]. Epigenetic mechanisms, such as DNA methylation, histone modification, chromatin remodelling and non-coding RNAs, change gene expression without altering the DNA sequence [5]. DNA is wrapped around histone bodies to form chromatin, and histone modifications influence chromatin availability as euchromatin (open and available for gene transcription) or heterochromatin (closed and less available for gene transcription) [6]. Epigenetic changes due to genetic or environmental factors can disrupt tightly regulated gene networks essential for brain development and immune function, contributing to NDDs [7,8].

Increasing evidence points to a transcriptomic signature in patients with NDDs of dysregulated ribosomal and immune pathways, which may be the result of chromatin dysfunction [7,8]. We hypothesise that therapeutic strategies targeting epigenetic/chromatin dysregulation may be a potential intervention for NDDs. Histone deacetylase (HDAC) enzymes remove acetyl groups from histones, resulting in chromatin condensation and transcriptional repression. Therefore, inhibiting these enzymes with HDAC inhibitors promotes an euchromatin structure, facilitating access for the transcriptional machinery and enhancing gene expression [9].

Butyrate is a short chain fatty acid derived from microbial fermentation of dietary fibre in the colon and has known HDAC inhibitory and anti-inflammatory properties [10]. We previously showed that the ketogenic diet, which increases the production of β-hydroxybutyrate, improved cognitive outcomes in a child with Kabuki syndrome (caused by *KMT2D* [a histone methyltransferase] mutation) [11]. Using single-cell RNA sequencing (scRNA-seq), we showed that ribosomal protein and immune pathways were dysregulated at baseline and normalised with a ketogenic diet in Kabuki syndrome. As both epigenetic and immune dysregulation are relevant to the pathophysiology of NDDs [[12], [13], [14], [15], [16]], butyrate is positioned as a promising therapeutic.

Due to the ubiquitous role of chromatin in cell function, *de novo* pathogenic variations in chromatin-related genes cause multiorgan genetic conditions including NDDs [11,[17], [18], [19]]. In this study, we first investigated gene expression of peripheral immune cells from children with NDDs, including those with *de novo* DNA variation in key chromatin-related genes: *KMT2D* (Kabuki syndrome)*, CHD7* (CHARGE syndrome), *MECP2* (Rett syndrome*)*, as well as patients with complex non-monogenic NDDs, using peripheral blood bulk RNA sequencing (RNA-seq). These genes (*KMT2D, CHD7, MECP2*) are central to chromatin function and are ubiquitously expressed in most cells of the body, including the immune system and brain. Next, we examined the effects of butyrate on gene regulation across these four conditions using scRNA-seq of peripheral mononuclear cells in patients at baseline, and after *in vitro* butyrate treatment. We used scRNA-seq due to the high statistical power generated by comparing 10,000s cells from n ​= ​1 comparisons. We hypothesised that *in vitro* butyrate treatment would modify gene expression and improve immune dysregulation in patients with NDDs.

## Methods

### Participants

19 children (<18 years of age) with functionally impairing NDDs were recruited to this study (median age 11 years, 14 females) (Table 1). 11 children (10 females) had *de novo* pathogenic variants in the chromatin-related genes *KMT2D* (n ​= ​4)*, CHD7* (n ​= ​2)*,* and *MECP2* (n ​= ​5)*,* while the remaining 8 children had NDDs with a history of loss of developmental skills (regression) but did not have a definable monogenic variant using trio exome sequencing (non-monogenic). None of the patients had an infection in the two weeks before blood draw. Table 1 briefly outlines the genetic variant and clinical phenotype of the children.

**Table 1:** Patients with Kabuki syndrome, CHARGE, Rett syndrome, and non-monogenic neuroregression recruited for bulk RNA seq and scRNA seq (scRNA seq denoted with ∗).

Gene, Syndrome	Age, sex	Variant	NDD	Other organ, details
*KMT2D*, Kabuki∗	9F	c.10077delG, p.(Gln3360Serfs∗32)	ID, ASD	Craniofacial, immune deficiency, hip dysplasia, scoliosis, short stature, precocious puberty
*KMT2D*, Kabuki	12M	c.12133C ​> ​T, p. (Gln4045∗)	Episodic cognitive decline, ADHD	Craniofacial, recurrent ear infection, immune deficiency, sub-aortic stenosis, short stature, hearing loss
*KMT2D*, Kabuki	17F	c.1301delT, p. (Leu434fs)	ASD, ADHD, ID	Craniofacial, recurrent pneumonia and ear infection, immune deficiency, aortic stenosis, scoliosis, short stature, hearing loss
*KMT2D*, Kabuki	19F	c.11607 dup, p. (Met3870Hisfs∗142)	ID, epilepsy	Craniofacial, hip dysplasia, scoliosis, short stature
*CHD7*, CHARGE∗	13F	c.7441C ​> ​T, p.(Gln2481∗)	ID, ASD, OCD, tics	Choanal atresia, coloboma, sensorineural deafness, congenital heart disease, recurrent infection, hip dysplasia
*CHD7*, CHARGE	14F	c.7727A ​> ​G, p.(Asp2576Gly)	ID	Bilateral coloboma, cleft palate, sensorineural hearing loss, renal tract issues, urinary tract infections, hypogonadotropic hypogonadism
*MECP2*, Rett∗	8F	c.473C ​> ​T, p.(Thr158Met)	ID, anxiety, stereotypy	Scoliosis, hip dyplasia Pneumonia, Feeding issues
*MECP2*, Rett	15F	c.1157_1197del41, p.(L386fs)	ID, stereotypy, epilepsy	Sleep disorder, breathing dysfunction, poor weight gain
*MECP2*, Rett	3F	c.473C ​> ​T, p.(Thr158Met)	ID, stereotypy, epilepsy	Hip dysplasia, scoliosis
*MECP2*, Rett	11F	c.808delC, p.(Arg270Glufs∗19)	ID, stereotypy, regression	Scoliosis, premature puberty
*MECP2*, Rett	7F	c.502C ​> ​T, p.(Arg168∗)	ID, stereotypy, epilepsy	Scoliosis, hip dysplasia
Nil∗	14M	–	ASD, Tourette syndrome, OCD, ADHD	Infection-provoked neuroregression
Nil	7M	–	ASD, ADHD, ID, OCD	Infection-provoked neuroregression
Nil	13M	–	ASD, ID, anxiety, depression	Infection-provoked neuroregression
Nil	8F	–	ASD, ADHD, OCD, anxiety	Infection-provoked neuroregression
Nil	14M	–	ASD, ID, Tourette, OCD, anxiety	Infection-provoked neuroregression
Nil	10F	–	ASD, ADHD, Tourette, OCD, anxiety	Autistic regression
Nil	5F	–	ADHD, OCD, tics, anxiety	Infection-provoked neuroregression
Nil	4F	–	Tourette, ADHD, OCD, anxiety	Infection-provoked neuroregression

Abbreviations: ADHD, attention deficit hyperactivity disorder; ASD, autistic spectrum disorder; ID, intellectual disability; OCD, obsessive-compulsive disorder. Syndromes marked with an asterisk (∗) were recruited for scRNA-seq.

### Control selection

We recruited 21 age- and sex-matched controls for comparison with the children with NDDs (median age 11 years, 15 females). The controls were healthy children who did not have neurodevelopmental or neuropsychiatric disorders, autoimmune diseases, severe allergic conditions, or infection in the previous two weeks before blood draw from the child.

### Sample collection

Venous blood from the participants was collected in Vacutainer ACD tubes (BD Biosciences, BD367756) after gaining written consent. Peripheral blood mononuclear cells (PBMCs) were isolated and stored, as previously described [11].

### Bulk RNA sequencing

RNA-seq was performed on whole blood from 19 patients and 21 controls by Australian Genome Research Facility (AGRF). This workflow included RNA extraction, depletion of ribosomal RNA via hybrid capture (Illumina Ribo-Zero), and Illumina TruSeq Stranded Total RNA Library Preparation (input 200-1,000 ​ng of Total RNA). The stranded RNA samples were sequenced on the Illumina NovaSeq X sequencing platform (2 ​× ​150 base pairs) for a depth of 50 million paired end reads. The cleaned sequence reads were aligned against the *Homo sapiens* genome (Build version hg38), and the STAR aligner (v2·5·3a) was used to map unique reads to the genomic sequences [20].

### Bioinformatics analysis of bulk RNA seq

RNA-seq data were analysed in the *R* statistical environment [21] using the *tidyverse* package [22]. Data preprocessing involved filtering and normalisation, followed by the removal of unwanted variation with the remove unwanted variation (*RUV*) package [23]. Differential expression analysis was performed using the *limma* package, with *p-*values calculated via the empirical Bayes (*eBayes*) method [24]. To control for multiple testing, *p-*values were adjusted using the false discovery rate (FDR) method, and genes with FDR <0.05 were considered significantly differentially expressed (DEGs).

We compared each NDD cohort (*KMT2D, CHD7, MECP2,* non-monogenic) with its own age-sex matched control group. Pathway enrichment analysis was performed via Over Representation Analysis (ORA) using the *clusterProfiler* package. DEGs were tested for pathway enrichment (FDR <0.05) using Gene Ontology (GO) terms. Dot plots of ORA GO results were plotted using *ggplot2* package. The top 5 upregulated and downregulated ORA GO non-simplified pathways were selected as representative GO terms and presented as bar charts and dot plots. For comparison, significant pathways were also shown across all other cohorts relative to controls in the dot plot, even if they were not among the top five in those cohorts. Quality control plots are presented in Supplementary Fig. 1.

### Treatment of PBMCs with butyrate

For *in vitro* investigation of butyrate's effects on gene expression, one child from each NDD cohort (*KMT2D, CHD7, MECP2*, non-monogenic) and two age- and sex-matched healthy controls were selected (denoted ∗ in Table 1). These samples were analysed using scRNA-seq, which enables profiling of 10,000s of cells per experiment and thereby provides high statistical power.

Frozen PBMC aliquots were thawed rapidly in a 37 ​°C water bath, followed by washing with thawing medium consisting of RPMI 1640 supplemented with 10 ​% foetal bovine serum (FBS), 1% GlutaMAX™, and 1% HEPES. The cells were then incubated in culture medium (RPMI 1640 supplemented with 10% FBS and 1% GlutaMAX™) at 37 ​°C in a 5% CO_2_ atmosphere for 3 ​h. Following the incubation, the cells were treated with 5 ​mM sodium butyrate (Thermo Fisher Scientific) in culture media or control culture media (untreated) for 24 ​h at 37 ​°C in a 5% CO_2_ atmosphere. A dose of 5 ​mM butyrate was chosen based on the ideal blood ketone ranges for the treatment of paediatric epilepsy with the ketogenic diet (2.0–5 ​mM β-hydroxybutyrate) [25] and butyrate has epigenetic effects at this concentration [11,26].

### HIVE™ single-cell RNA sequencing

Following the 24-h incubation with butyrate or control culture media, the cells were harvested from the wells by scraping with a 1 ​mL pipette tip, washed, and stained with DAPI (1:100). The live cells (DAPI-negative) were sorted by the Westmead Institute for Medical Research (WIMR) using the BD FACSAria™ III Cell Sorter and subsequently loaded into HIVE™ devices, following the manufacturer's instructions [27]. 12 HIVE™ devices were loaded with approximately 30,000 ​cells from each sample (individual butyrate-treated and untreated samples from 4 patients and 2 controls) in 1 ​mL of DPBS ​+ ​1% FBS followed by 3 ​mL of cell media (DPBS ​+ ​1% FBS).

HIVEs™ were then placed onto a spin plate and spun at 30 × *g* for 3 ​min to allow the single-cells to settle into picowells containing 3’ transcript-capture beads. The solution was removed from the HIVE™ devices, and 2 ​mL sample wash solution (provided in kit) was added to each. The sample wash solution was removed, and the cell-loaded HIVEs™ were frozen at −80 ​°C after the addition of 2 ​mL cell preservation solution (provided in kit), before being transferred to Australian Genome Research Facility (AGRF Ltd, Westmead) and processed through to single-cell NGS libraries [27]. Following standard protocol, cell-loaded HIVE™ devices were sealed with a semi-permeable membrane, allowing for the use of the strong lysis solution followed by the addition of hybridisation solution. After collection, beads with captured transcripts were extracted from the HIVE™ device by centrifugation. The remaining HIVE™ library preparation steps were conducted in a 96-well plate format [28]. The size distribution and quality of the final libraries were determined on a TapeStation 2000 platform with HD5000 ScreenTape System (Agilent Technologies, Santa Clara, CA, USA). The concentration of final pooled libraries was determined by qPCR. HIVE™ scRNA-seq libraries were sequenced using specific primers contained in the kit on an Illumina® NovaSeq® X platform, as previously described (AGRF Ltd, Melbourne) [8].

### Single-cell RNA sequencing bioinformatic analysis

Normalisation was performed using SCTransform in *Seurat Package* (Version 5) and immune cell types were assigned with scType and scPred. We focused on broad immune cell types including T cells, B cells monocytes, eosinophils, myeloid dendritic cells and natural killer cells. Interestingly, scType results suggest that butyrate has a unique effect on CD4 T cell transcriptomes, suppressing expression of immune genes often used in scRNA-seq to identify CD4 T cells. We therefore clustered CD4 and CD8 T cells together as one cell population, rather than separating the populations for analysis. Merged data were then split by cell type and separately normalised, scaled, and integrated between patients using *harmony*, then UMAPs (uniform manifold approximation and projection) were made using the first 30 PCA dimensions. DEGs were identified using *FindMarkers.* Pathway enrichment analysis was the same as used in RNA-seq method. Bar and dot plots of ORA GO results were plotted using *ggplot2* package. We analysed comparisons with all cells (bulk) as well as with the T cell population only. T cells were the largest individual cell population across our samples, representing 52–83% of the cell population per sample. Other cell populations were too small to analyse separately.

To investigate the system-wide effects of butyrate, functional network grouping was conducted using a similarity matrix generated through the *pairwise_termsim* function and visualised with the *emapplot* function from the *enrichplot* package. The top 10 enriched GO terms from each ontology (Biological Process, Molecular Function, and Cellular Component) were organised into a network, with edges representing the overlap between gene sets, and plotted onto an enrichment map.

### Ethics approval

Ethical approval was granted by the Sydney Children's Hospitals Network Human Research Ethics Committee (HREC/18/SCHN/227, 2021/ETH00356). All families provided written informed consent for the study.

## Results

First, we explored the baseline gene expression in the 4 groups using RNA-seq compared to age-sex matched controls.

### Peripheral blood bulk RNA-seq: baseline patient vs control

#### KMT2D (Kabuki syndrome)

**Fig. 1:**
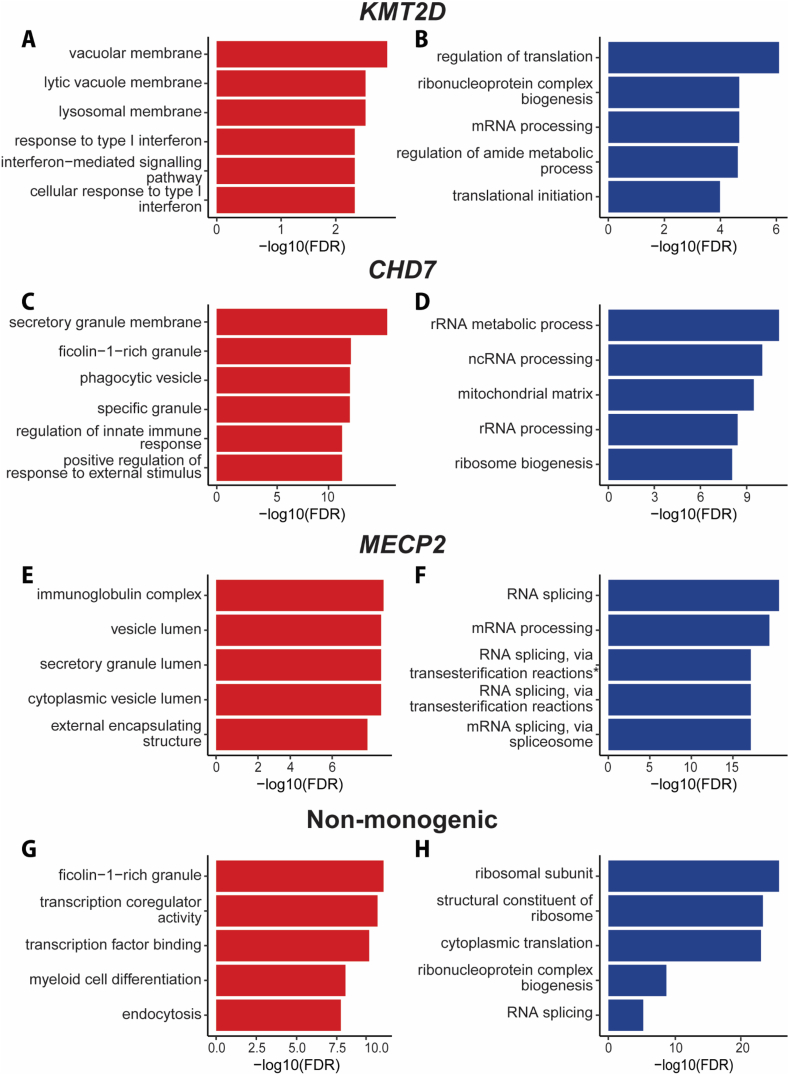
**Top five up- (red) and down-regulated (blue) ORA GO pathways in bulk RNA sequencing of whole blood from NDD cohorts versus controls.** The level of pathway enrichment is determined by -log10(FDR), where significant pathways have an FDR <0.05. The comparison of *KMT2D* cohort (n ​= ​4) versus controls (n ​= ​18) is presented as bar plots for **(A)** the top 5 upregulated ORA GO pathways (red), including immune and cellular function pathways, and **(B)** the top 5 downregulated ORA GO pathways (blue) including ribosomal and translation pathways. The comparison of *CHD7* cohort (n ​= ​2) versus controls (n ​= ​14) is presented as bar plots for **(C)** the top 5 upregulated ORA GO pathways (red), including immune and cellular function pathways, and **(D)** the top 5 downregulated ORA GO pathways (blue) including ribosomal and mitochondrial pathways. The comparison of *MECP2* cohort (n ​= ​5) versus controls (n ​= ​14) is presented as bar plots for **(E)** the top 5 upregulated ORA GO pathways (red), including immune and cellular function pathways, and **(F)** the top 5 downregulated ORA GO pathways (blue) including RNA pathways. The comparison of non-monogenic NDD cohort (n ​= ​8) versus controls (n ​= ​8) is presented as bar plots for **(G)** the top 5 upregulated ORA GO pathways (red), including immune and cellular function pathways, and **(H)** the top 5 downregulated ORA GO pathways (blue) including ribosomal and translation pathways.

*KMT2D* encodes a lysine (histone) methyltransferase called MLL2 which is required for di- and tri-methylation of histone 3 lysine 4, central to histone methylation and chromatin arrangement [29]. Thus, pathological variants in *KMT2D* disrupt this mechanism, promoting a closed chromatin structure and limiting gene transcription [30]. Using RNA-seq we compared Kabuki syndrome (*KMT2D*, n ​= ​4*,* median age 14.5 years, 3 female) with age-sex matched controls (n ​= ​18, median age 11 years, 14 female) and found that upregulated GO pathways were immune and cellular function pathways, and downregulated GO pathways were ribosomal and translation pathways (Fig. 1A and B).

### *CHD7* (CHARGE syndrome)

The *CHD7* gene encodes the chromodomain helicase DNA binding protein 7, which is involved in regulating gene expression through chromatin remodelling [31]. Using RNA-seq we compared patients with CHARGE syndrome (*CHD7,* n ​= ​*2,* median age 13.5 years, both female) with age-sex matched controls (n ​= ​14, median age 9 years, both female), and found that upregulated GO pathways were immune and cellular function pathways, and the downregulated GO pathways were primarily RNA and ribosomal pathways (Fig. 1C and D).

### *MECP2* (Rett syndrome)

The *MECP2* gene encodes the methyl-CpG binding protein 2 (MeCP2), which is a transcription factor and chromatin-associated protein [32,33]. Using RNA-seq we compared patients with Rett syndrome (*MECP2,* n ​= ​5, median age 8.5 years, all female) with age-sex matched controls (n ​= ​14, median age 9 years, all female), and found the top five upregulated GO pathways were immune and cellular function pathways, and the downregulated GO pathways were ribosomal and RNA pathways (Fig. 1E and F).

### Non-monogenic NDD

The non-monogenic group had complex NDDs including neuroregression (Table 1). Using RNA-seq we compared patients with non-monogenic NDD (n ​= ​8, median age 9 years, 4 female) with age-sex matched controls (n ​= ​8, median age 12.5 years, 4 female) and found the top 5 upregulated GO pathways were immune and transcription pathways, and the downregulated pathways were ribosomal and RNA pathways (Fig. 1G and H).

### Peripheral blood RNA-seq: common gene dysregulation across patient cohorts

**Fig. 2:**
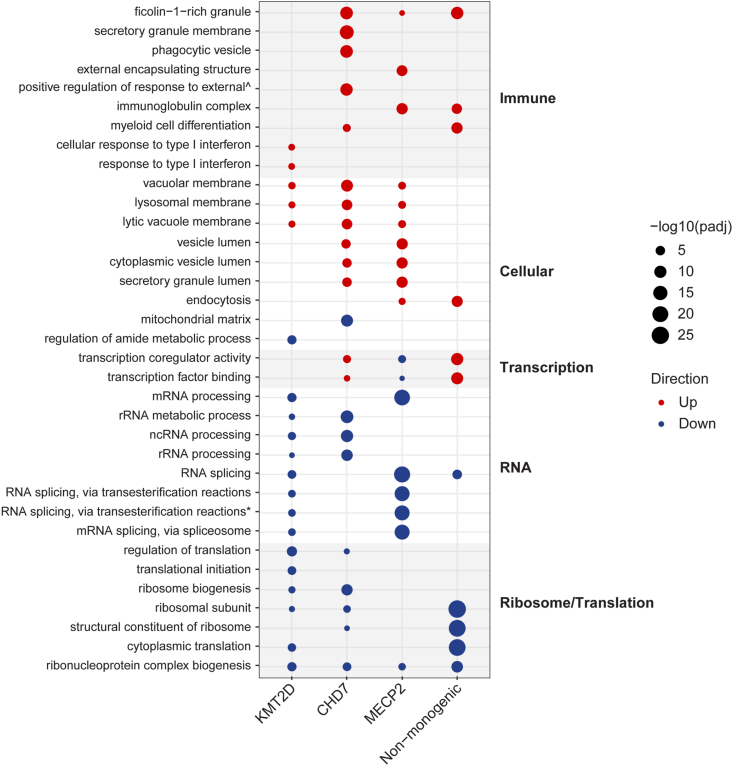
**Dot plot visualising the top 5 upregulated and downregulated ORA GO pathways in bulk RNA sequencing of whole blood from NDD cohorts versus controls.** The top 5 upregulated and downregulated ORA GO pathways for each cohort were selected as representative GO terms. For comparison, significant pathways were also shown across all other cohorts relative to controls in the dot plot, even if they were not among the top five in those cohorts. Each cohort is labelled on the axis as *KMT2D* (n ​= ​4)*, CHD7* (n ​= ​2)*, MECP2* (n ​= ​5) or non-monogenic (n ​= ​8), and are compared to their respective healthy matched control groups (n ​= ​18, n ​= ​14, n ​= ​14, n ​= ​8, respectively). Red dots represent upregulated pathways, and blue dots represent downregulated pathways. The size of the dot represents the significance of pathway enrichment determined by -log10(padj) value, where padj is the adjusted *p* value. Enriched pathways are labelled on the left-hand y-axis. The pathways are clustered together based on similar function and then ranked according to enrichment. The pathways clusters are indicated by shading on the dot plot and labels to the right of the dot plot.

To explore common molecular dysregulation, we took the top 5 upregulated and top 5 downregulated pathways from each of the 4 patient groups versus control comparisons (Fig. 2). There were common themes with upregulated immune and cellular pathways, and downregulated RNA and ribosomal pathways. The common significantly downregulated pathway across all NDD groups versus controls was the ribonucleoprotein complex biogenesis (Fig. 2). There were 19 downregulated DEGs from this pathway that were common across the NDD cohorts, which can be clustered into processes related to ribosome biogenesis, RNA binding, rRNA processing, RNA modification, translation initiation, mRNA processing and exosome (Table 2).

**Table 2:** Common DEGS (n ​= ​19) across *KMT2D, CHD7, MECP2* and non-monogenic NDD groups from the downregulated ribonucleoprotein complex biogenesis pathway.

	Gene	Additional & Associated Gene Functions
*Ribosome biogenesis and rRNA processing*	*BRIX1*	Ribosomal LSU assembly
	*MAK16*	5.8S and LSU-rRNA maturation
	*NOL11*	SSU rRNA maturation; transcription regulation
	*PWP1*	Histone chaperone; chromatin organisation; LSU rRNA transcription regulation
	*UTP15*	SSU rRNA maturation; transcription regulation (RNA Pol I); SSU processome
	*UTP23*	SSU rRNA maturation; SSU processome
	*RRP15*	5.8S and LSU-rRNA maturation; LSU precursor complex
	*RPF1*	5.8S and LSU-rRNA maturation
	*RIOK2*	Atypical kinase; SSU rRNA maturation and export; SSU processome
*RNA metabolism*	*DDX1*	ATP-dependent RNA helicase; transcription and translation initiation regulation; spliceosome regulation; chromatin binding
	*DDX18*	ATP-dependent RNA helicase; LSU rRNA maturation
*RNA modification*	*METTL5*	Methyltransferase; translation regulation
*mRNA processing/exosome*	*CPSF6*	RNA and mRNA binding; ribosomal LSU binding; mRNA processing, cleavage and polyadenylation;
	*NCBP1*	mRNA capping; mRNA splicing/export; translation regulation
	*EXOSC1*	RNA exosome component; RNA/rRNA processing
	*LYAR*	Transcription regulation (RNA Pol I/II)
*Translation initiation and regulation*	*EIF2A*	Translation initiation factor; ribosome assembly; start codon recognition
	*NGDN*	Translation regulation (also known *EIF4E)*; SSU processome
	*ABCE1*	Ribonucleoside triphosphate phosphatase activity; ribosomal SSU binding; translation initiation and regulation; rescue of stalled ribosome; ribosome disassembly

Genes were organised into groups (left column) using OpenAI's ChatGPT. Information in ‘Additional and Associated Gene Functions’ column were found on the National Centre for Biotechnology Information's Gene Database. Abbreviations: ATP, adenosine triphosphate; LSU, large subunit; mRNA, messenger ribonucleic acid; SSU, small subunit; RNA, ribonucleic acid; rRNA, ribosomal ribonucleic acid; RNA pol I/II, RNA polymerase I/II.

### Single-cell RNA sequencing (scRNA-seq): differentially expressed genes

A total of 101,539 ​cells were sequenced across 12 samples from four patients with different NDDs and two matched healthy controls at baseline (untreated, media only) and after *in vitro* butyrate treatment (media + 5 ​mM butyrate). UMAP analysis of biological samples revealed 6 distinct cell clusters (Supplementary Fig. 2). We compared ‘untreated patient’ versus ‘untreated control’ to determine the ‘baseline’ abnormality, and ‘butyrate-treated patient’ versus ‘untreated patient’, to determine the effect of butyrate on patient cells. These comparisons yielded 592 to 6909 DEGs per comparison in the bulk cell analysis, and 175 to 2229 DEGs per comparison in the T cell analysis (Supplementary Table 1).

### Single cell RNA sequencing: baseline findings and effects of *in vitro* butyrate treatment on PBMCs

**Fig. 3:**
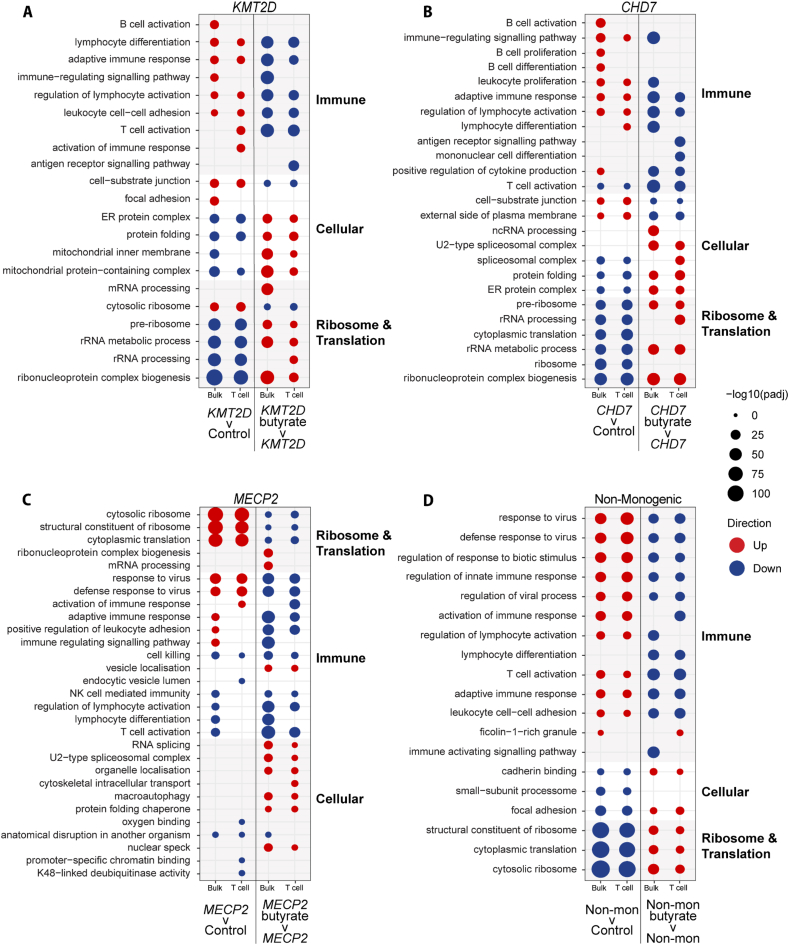
**Dot plots visualising the top 10 upregulated and downregulated ORA GO pathways in untreated patient versus control PBMCs, and butyrate-treatedversus untreated patient PBMCs.** The top 10 upregulated and downregulated ORA GO pathways for T cells and bulk cells were selected as representative GO terms and are presented on the dot plots. Both bulk analysis and T cell analysis are included, as labelled on the x-axis. The left-hand columns present the untreated patient versus control pathways, and the right-hand columns present the butyrate-treated versus untreated patient pathways. Red dots represent upregulated pathways, and blue dots represent downregulated pathways. The size of the dot represents the significance of pathway enrichment determined by -log10(padj) value, where padj is the adjusted *p* value. The pathways are clustered together based on similar function and then ranked according to enrichment. The pathway clusters are indicated by shading on the dot plot and labels to the right of the dot plot. There are separate plots for each NDD patient: **(A)***KMT2D* (n ​= ​1), **(B)***CHD7* (n ​= ​1), **(C)***MECP2* (n ​= ​1), **(D)** Non-monogenic (n ​= ​1).

Using incubation of PBMCs with butyrate followed by scRNA-seq, we present the similarities and differences in selected individuals with *KMT2D* (Fig. 3A), *CHD7* (Fig. 3B), *MECP2* (Fig. 3C), and non-monogenic NDD (Fig. 3D) at baseline (case vs control) and after butyrate treatment (case-butyrate vs case) (Fig. 3A–D).

At baseline, both bulk and T cell comparisons were similar to the RNA-seq data (Fig. 1, Fig. 2 and Supplementary Figs. 3 and 4), with ribosome pathways downregulated and immune pathways upregulated in the *KMT2D* (Supplementary Figs. 3A and 4A)*, CHD7* (Supplementary Figs. 3B and 4B) and non-monogenic NDD (Supplementary Figs. 3D and 4D) PBMCs, whereas these pathways were more mixed in the *MECP2* PBMCs (Supplementary Figs. 3C and 4C).

Exploring the effects of butyrate, the baseline upregulated immune pathways in *KMT2D* (Fig. 3A)*, CHD7* (Fig. 3B) and non-monogenic (Fig. 3D) patients were downregulated after butyrate treatment. The baseline downregulated ribosomal and rRNA processing pathways in *KMT2D* (Fig. 3A)*, CHD7* (Fig. 3B) and non-monogenic (Fig. 3D) cells were upregulated after butyrate treatment. A similar effect was demonstrated when healthy control PBMCs were incubated with butyrate: immune pathways were downregulated, and ribosomal pathways were upregulated (Supplementary Fig. 5).

By contrast, for the selected *MECP2* patient (Fig. 3C)*,* there was less consistent modification of pathways, although the baseline upregulated ribosomal pathways and dysregulated immune pathways were generally downregulated by butyrate.

### Systems-wide effects of butyrate treatment

**Fig. 4:**
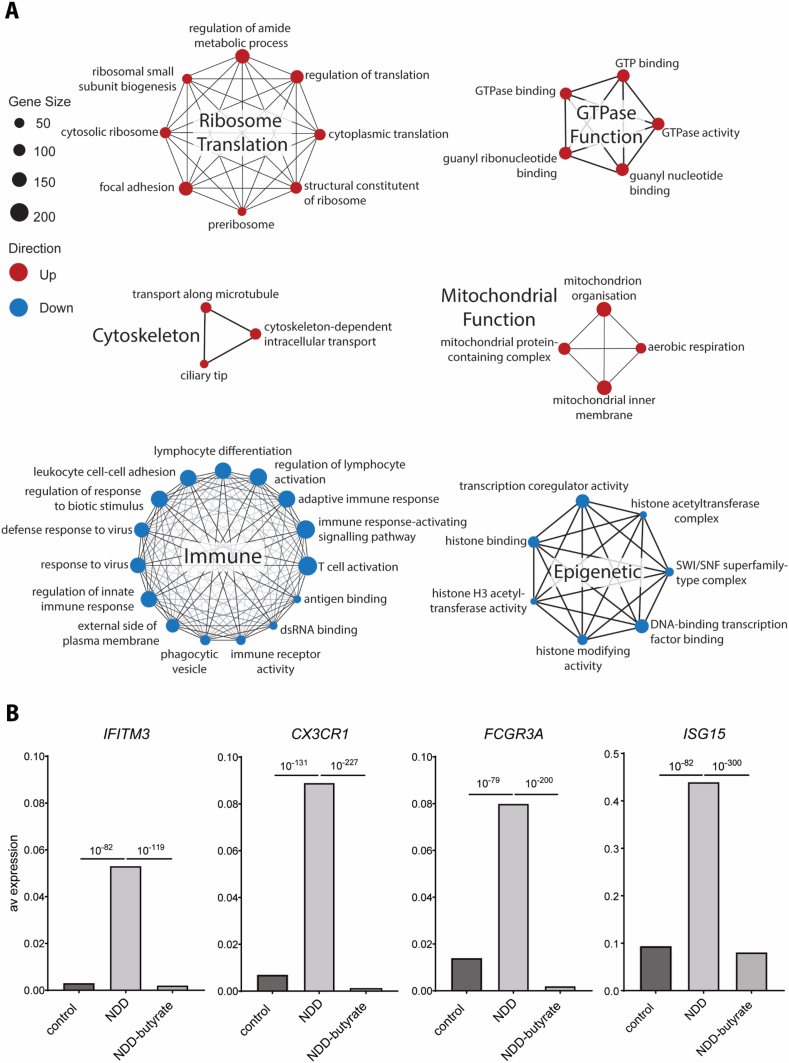
**System wide effects of butyrate treatment on PBMCs from the selected non-monogenic NDD case. (A)** Functional network groupings of top 10 enriched GO terms from each ontology (biological process, molecular function and cellular component) in butyrate-treated versus untreated PBMCs from the non-monogenic patient comparison. Gene set size (number of genes) is represented by the size of the dot and edges represent the overlap between gene sets. Upregulated GO pathways are represented in red and downregulated GO pathways are represented in blue. **(B)** Comparison of average expression of *IFITM3, CX3CR1, FCGR3A, ISG15* immune genes between control PBMCs, NDD patient PBMCs and butyrate-treated NDD patient PBMCs. Statistical significance (*p* value) is indicated as line above graphs. Note difference in y-axis for *ISG15*.

Next, we explored the system-wide effects of butyrate treatment on the non-monogenic PBMCs (bulk analysis) (Fig. 4A). Butyrate treatment (non-monogenic butyrate vs non-monogenic) upregulated pathways related to ribosome/translation, GTPase function, cytoskeleton, and mitochondrial function, and downregulated epigenetic (histone, transcription activity) and immune pathways. Furthermore, we found that butyrate treatment normalised immune-related gene expression of important selected immune genes *IFITM3, CX3CR1, ISG15 and FCGR3A* in patient cells (Fig. 4B).

## Discussion

Epigenetic mechanisms are increasingly recognised as key contributors to NDDs, arising from *de novo* pathogenic DNA variations in chromatin-related genes or through the interplay of common gene variants and environmental factors [7,8,11,19,34]. We selected children with *de novo* mutations in key chromatin-related genes *KMT2D* (Kabuki syndrome)*, CHD7* (CHARGE syndrome) and *MECP2* (Rett syndrome) as models of chromatin dysregulation, as well as children with non-monogenic NDDs who had suffered periods of neuroregression. Using RNA-seq, we observed that *KMT2D, CHD7*, *MECP2* and non-monogenic NDD cells showed similar transcriptional changes at baseline, characterised by downregulated ribosomal and upregulated immune pathways.

Whilst Kabuki and CHARGE syndrome are clinically distinct, there is significant phenotypic overlap [31,35,36]. CHD7 (encoded by *CHD7*) and MLL2 (encoded by *KMT2D*) interact with the same transcriptional machinery [31], suggesting shared regulatory pathways that may account for their similar transcriptomic profiles seen in our study. Additionally, dysregulation of ribosomal and immune pathways in non-monogenic NDD cells is a common RNA-seq signature that we and others have observed in previous NDD cohorts [7,8,34,37,38]. This finding suggests gene regulation, or specific chromatin dysregulation, may be a common feature of non-monogenic NDDs. Although the findings across different syndromes presented significant similarities, the *MECP2* cells showed more variable pathway regulation, possibly due to the multifunctional nature of MeCP2 (encoded by *MECP2*) as both a repressor and promoter of gene transcription [33]. We hypothesise that chromatin dysregulation is an important mechanism across NDDs and is associated with an RNA-seq signature characterised by ribosomal and immune dysregulation. We found the pathway ‘ribonucleoprotein complex biogenesis’ was downregulated in all groups, and the common downregulated genes are central to RNA processing and ribosomal function.

Ribosomal gene expression and biogenesis are tightly regulated by epigenetic mechanisms such as histone modifications, linking chromatin state to the cell's capacity for protein synthesis [39]. *CHD7* is a positive regulator of ribosomal RNA (rRNA) biogenesis. Depleting cells of *CHD7* results in hypermethylation of the rDNA promoter, and reduction of 45S pre-rRNA levels, cell proliferation, and protein synthesis [40]. As a histone modifier, *KMT2D* regulates ribosomal gene transcription and translation, and its depletion or mutation disrupts ribosomal protein biogenesis [11,41]. Ribosomes are the site of mRNA translation and play a critical role in brain development and disruptions to their translational machinery are associated with NDDs [42]. Perturbations in ribosomal biogenesis can impair protein synthesis essential for dendritic growth and synaptic function, leading to altered neuronal connectivity characteristic of NDDs [42]. Environmental exposures such as infections and oxidative stress, can further disrupt ribosome production and function, compromising cellular homeostasis [[43], [44], [45]].

Due to the central role of chromatin regulation in determining cell fate, Kabuki syndrome, CHARGE syndrome, and Rett syndrome share common features of multiorgan involvement, in addition to neurodisability [42,46]. KMT2D, CHD7 and MeCP2 expression in immune cells also predisposes affected children to recurrent infections and we have observed infection-triggered neurodevelopmental or neuropsychiatric regression in these patients [19]. In Kabuki syndrome, there is increased susceptibility to infection in up to 70 ​% of patients, due to B cell dysfunction, hypogammaglobulinemia, and CD4^+^ T cell deficiency [47,48]. In CHARGE syndrome due to *CHD7* mutations, immune deficiency is variable but includes T cell lymphopenia, B cell dysfunction, hypogammaglobulinemia, or rare severe combined immunodeficiencies [48,49]. Rett syndrome, caused by *MECP2* mutations, is linked to elevated proinflammatory cytokines, altered microglia and macrophage activation [50,51]. Together, these findings highlight the role of chromatin-related gene dysfunction in driving both neurodevelopmental and immune abnormalities, emphasising the importance of epigenetic regulation in maintaining neuroimmune homeostasis in NDDs.

Butyrate, a short-chain fatty acid and HDAC-inhibitor with anti-inflammatory properties [52,53], reversed the abnormalities observed at baseline in our *in vitro* scRNA-seq comparisons. We used scRNA-seq given the statistical power of analysing 10,000+ individual cells in n ​= ​1 comparisons. Pathway analysis revealed butyrate-induced upregulation of ribosome/translation, GTPase activity, cytoskeletal organisation, mitochondrial pathways, and downregulation of epigenetic (histone, transcription activity) and immune pathways. Similar findings were observed in the control cells treated with butyrate.

Butyrate suppresses inflammation by promoting regulatory T cells, inhibiting pro-inflammatory T cells and macrophages, and inducing tolerogenic dendritic cells through HDAC inhibition and downstream signalling [54,55]. Through its influence on the gut–brain axis, butyrate modulates brain function via vagal nerve activation, endocrine signalling, and immune pathways [56]. Butyrate crosses the blood-brain barrier and inhibits HDACs, increasing histone acetylation, chromatin accessibility, and neuroprotective gene expression [57]. These effects promote hippocampal neurogenesis, synaptic plasticity, and memory formation [57]. Butyrate also modulates glial cell function, by promoting an anti-inflammatory phenotype, suppressing NF-κB–driven pro-inflammatory cytokines, supporting the resolution of neuroinflammation [58].

Given that *de novo* pathogenic DNA variations in chromatin-related genes account for only a minority of NDDs, it was clinically important to investigate how butyrate could be applied more broadly to non-monogenic NDDs. We observed that butyrate upregulated the expression of ribosome translation, GTPase function, cytoskeleton and mitochondrial function, and downregulated immune and epigenetic pathways in the cells of the non-monogenic NDD patient. GTPase activity and cytoskeleton organisation pathways are important mechanisms of chromatin remodelling [59]. GTPases play critical roles in regulating chromatin structure and transcription by controlling actin dynamics and the localisation of chromatin modifiers [[60], [61], [62]]. Cytoskeletal organisation further influences chromatin accessibility by altering nuclear shape and tension [63]. The observed downregulation of epigenetic pathways, including histone binding and histone modifying activity, was likely due to butyrate's previously described HDAC inhibitory function [54,55,57]. Based on our findings, butyrate demonstrates promising epigenetic, anti-inflammatory and immunomodulatory effects, supporting its potential as a therapeutic intervention for NDDs.

Clinically, the ketogenic diet represents the most established analogue of butyrate, as it generates the ketone body β-hydroxybutyrate [64]. However, adherence to the ketogenic diet is challenging due to the need for strict dietary compliance and medical monitoring of ketones [65]. Exogenous ketone supplementation has emerged as a more practical alternative, with ketone salts (e.g., sodium, potassium, and calcium butyrate), esters (e.g., d-β-hydroxybutyrate isoform), and monoesters ((*R)*-3-hydroxybutyl *(R)*-3-hydroxybutyrate) now commercially available [66,67]. These compounds can acutely elevate circulating β-hydroxybutyrate levels without requiring prolonged fasting or carbohydrate restriction [67]. Nonetheless, they are limited by poor bioavailability, first-pass hepatic metabolism, and a short half-life, necessitating multi-dosing [53]. Importantly, most evidence for their efficacy is derived from acute, single-dose studies conducted under controlled laboratory conditions [66,68], and the long-term, clinically relevant effects of sustained exogenous ketone use remain underexplored [69]. To address the limitations of conventional supplementation, various modified butyrate derivates have been developed to enhance bioavailability. These include esterified or starch-conjugated forms such as serine-butyrate [70,71], high-amylose maize-resistant starch modified with acetate and butyrate (HAMSAB) [72], and N-(1-carbamoyl-2-phenyl-ethyl) butyramide (FBA) [73]. Further studies are required to evaluate the therapeutic efficacy, optimal dosing, and HDAC-inhibitory potential of butyrate and its derivates in human NDDs.

Limitations of this study include the use of PBMCs rather than brain tissue. However, due to the ubiquitous nature of chromatin in most cell types, RNA-seq of whole blood and scRNA-seq of PBMCs make valuable and feasible potential biomarkers [11,39,74,75]. Some of the groups had small size (n ​= ​2 for *CHD7*), reducing the generalisability of our findings; however, we observed generally consistent findings across RNA-seq and scRNA-seq, supporting the reproducibility of our baseline data. Another limitation of our study is that we focused on bulk and the larger cell population (T cells) for our scRNA-seq analysis. The other cell populations (B cells, monocytes, natural killer cells, etc.) were too small to yield statistically powerful results across the comparison conditions. Additionally, targeted epigenetic analysis, ATAC sequencing, histone modification profiling, and methylation analysis, are needed to explore the role of epigenetics in NDDs and the impact of butyrate. Detailed functional immune testing is also required to confirm cellular immune deficits in NDDs, and their modification by butyrate. Future randomised clinical trials and *in vivo* studies in larger cohorts, including different doses and duration, are needed to evaluate the safety, therapeutic efficacy, and biological impact of butyrate in improving symptoms of NDDs.

Overall, our study shows that children with NDDs, both with and without DNA variants in chromatin-related genes, share transcriptional abnormalities in peripheral immune cells. We propose that chromatin dysregulation leads to a common RNA-seq signature involving ribosomal pathways with associated immune dysregulation. Chromatin dysregulation may represent a common mechanism across NDDs, affecting both brain and immune cells [7,8,11,34]. *In vitro* treatment with butyrate largely reversed these gene expression abnormalities, highlighting its potential as a therapeutic modulator of epigenetic and immune dysfunction in NDDs.

## Resource availability

Anonymised data not published within this article will be made available by request from any qualified investigator. scRNA-seq data was uploaded to https://www.ncbi.nlm.nih.gov/geo/query/acc.cgi?acc=GSE296038.

## Author Contributions

HN, BK and RCD conceptualised and designed the experiments. JH, VXH, HN, ET, XL, RD, BK and SP performed the experiments and data acquisition. VXH and BG performed bioinformatic analysis. JH, VXH, HN, ET, MW, CE, BK, SM, MH, PV, SP, RCD analysed and interpreted the data. JH, VXH and RCD wrote the original manuscript. All authors critically revised and edited the manuscript. All authors approved the final version of the article.

## Funding

This study was funded by NHMRC Investigator (Dale) 1193648, Petre Foundation, NFMRI and Avant translational grant.

## Declaration of competing interest

The authors declare the following financial interests/personal relationships which may be considered as potential competing interests: Russell Dale reports financial support was provided by National Health and Medical Research Council. Russell Dale reports financial support was provided by Petre Foundation. Russell Dale reports financial support was provided by National Foundation for Medical Research and Innovation. Russell Dale reports financial support was provided by Avant Foundation. If there are other authors, they declare that they have no known competing financial interests or personal relationships that could have appeared to influence the work reported in this paper.

## Appendix A Supplementary data

Supplementary Fig. 1 **Quality Control Plots from bulk RNA-seq of whole blood from NDD and control cohorts.** Principal component analysis (PCA) plots show the distinct clustering of samples from the patient cohorts (fuchsia) and control cohorts (blue). Bar charts present the total number of sequencing reads in millions for each sample. For the *KMT2D* cohort (fuchsia) versus control cohort (blue), **(A)** presents the PCA plot and **(B)** presents the total number of reads for each sample. For the *CHD7* cohort (fuchsia) versus control cohort (blue), **(C)** presents the PCA plot and **(D)** presents the total number of reads for each sample. For the *MECP2* cohort (fuchsia) versus control cohort (blue), **(E)** presents the PCA plot and **(F)** presents the total number of reads for each sample. For the non-monogenic NDD cohort (fuchsia) versus control cohort (blue), **(G)** presents the PCA plot and **(H)** presents the total number of reads for each sample. The non-monogenic samples are labelled as “nonmono” on the y-axis. **Supplementary Fig. 2 UMAP and Quality Control Plots from scRNA-seq of NDD case and control PBMCs. (A)** Uniform manifold approximation and projection (UMAP) analysis of 12 samples (including 4 children with monogenic and non-monogenic NDDs and 2 healthy controls) in scRNA-seq identified 6 distinct cell clusters including B cells (light blue), classical monocytes (grey), eosinophils (orange), myeloid dendritic cells (gold), natural killer cells (dark blue), and T cells (aqua), **(B)** Proportion of individual cell types across samples. **(C)** Heat map of the expression of identifying cell-surface markers for each cell cluster. Quality of samples is presented in the following violin plots: **(D)** Number of features, **(E)** Number of counts, and **(F**) mitochondrial percentage per sample. The non-monogenic samples are labelled as “nonmono” on the y-axes. **Supplementary Fig.3 Single-cell RNA sequencing of PBMCs from selected NDD cases versus control: bulk cell analysis of top 5 ORA GO pathways.** The top 5 upregulated (red) and top 5 downregulated (blue) ORA GO pathways for each comparison are presented on bar plots. The significance of enrichment of the pathways is determined by -log10(padj), where padj is the adjusted p value. Note the different values on the x-axes. For *KMT2D* versus control, **(A)** presents the top 5 upregulated pathways, including adhesion, immune and ribosomal pathways, and **(B)** presents the top 5 downregulated pathways including ribosomal and mitochondrial pathways. For *CHD7* versus control, **(C)** presents the top 5 upregulated pathways, including immune pathways, and **(D)** presents the top 5 downregulated pathways including ribosomal and rRNA pathways. For *MECP2* versus control, **(E)** presents the top 5 upregulated pathways, including immune and ribosomal pathways, and **(F)** presents the top 5 downregulated pathways, including immune pathways. For the non-monogenic NDD case versus control, **(G)** presents the top 5 upregulated pathways, including immune pathways, and **(H)** presents the top 5 downregulated pathways, including ribosomal pathways. All comparisons are n ​= ​1. **Supplementary Fig. 4 Single-cell RNA sequencing of PBMCs from selected NDD cases versus control: T cell analysis of top 5 ORA GO pathways.** The top 5 upregulated (red) and top 5 downregulated (blue) ORA GO pathways for each comparison are presented on bar plots. The significance of enrichment of the pathways is determined by -log10(padj), where padj is the adjusted *p* value. Note the different values on the x-axes. For *KMT2D* versus control, **(A)** presents the top upregulated pathways including adhesion, immune and ribosomal pathways, and **(B)** presents the top downregulated, including ribosomal pathways. For *CHD7* versus control, **(C)** presents the top upregulated pathways, including immune pathways, and **(D)** presents the top downregulated pathways, including ribosomal and rRNA pathways. For *MECP2* versus control, **(E)** presents the top upregulated pathways, including immune and ribosomal pathways, and **(F)** and the top downregulated pathways including protein regulation, cytoskeletal, metabolic, gene regulation, and immune pathways. For the non-monogenic case versus control **(G)** presents the top upregulated pathways including immune pathways, and **(H)** presents the top downregulated pathways including ribosomal pathways. All comparisons are *n* ​= ​1. **Supplementary Fig. 5 Single-cell RNA sequencing of butyrate-treated control PBMCs compared to untreated control PBMCs.** The top 10 upregulated (red) and top 10 downregulated (blue) ORA GO pathways for butyrate-treated (n ​= ​1) versus untreated (n ​= ​1) control PBMCs (female control) presented on bar plots. The significance of enrichment of the pathways is determined by -log10(padj), where padj is the adjusted *p* value. **(A)** presents the upregulated pathways including ribosome, mitochondrial and cellular function pathways and **(B)** presents the downregulated pathways, including immune pathways. Supplementary Fig. 1
