# Supplementary material for: Butyrate modifies epigenetic and immune pathways in peripheral mononuclear cells from children with neurodevelopmental disorders associated with chromatin dysregulation
Source: Neurotherapeutics. 2025 Nov 19;23(1):e00792. doi: 10.1016/j.neurot.2025.e00792 (PMC12976508; doi:10.1016/j.neurot.2025.e00792)
Supplement: Supplementary Fig. 1 — Quality Control Plots from bulk RNA-seq of whole blood from NDD and control cohorts. Principal component analysis (PCA) plots show the distinct clustering of samples from the patient cohorts (fuchsia) and control cohorts (blue). Bar charts present the total number of sequencing reads in millions for each sample. For the KMT2D cohort (fuchsia) versus control cohort (blue), (A) presents the PCA plot and (B) presents the total number of reads for each sample. For the CHD7 cohort (fuchsia) versus control cohort (blue), (C) presents the PCA plot and (D) presents the total number of reads for each sample. For the MECP2 cohort (fuchsia) versus control cohort (blue), (E) presents the PCA plot and (F) presents the total number of reads for each sample. For the non-monogenic NDD cohort (fuchsia) versus control cohort (blue), (G) presents the PCA plot and (H) presents the total number of reads for each sample. The non-monogenic samples are labelled as “nonmono” on the y-axis. Supplementary Fig. 2 UMAP and Quality Control Plots from scRNA-seq of NDD case and control PBMCs. (A) Uniform manifold approximation and projection (UMAP) analysis of 12 samples (including 4 children with monogenic and non-monogenic NDDs and 2 healthy controls) in scRNA-seq identified 6 distinct cell clusters including B cells (light blue), classical monocytes (grey), eosinophils (orange), myeloid dendritic cells (gold), natural killer cells (dark blue), and T cells (aqua), (B) Proportion of individual cell types across samples. (C) Heat map of the expression of identifying cell-surface markers for each cell cluster. Quality of samples is presented in the following violin plots: (D) Number of features, (E) Number of counts, and (F) mitochondrial percentage per sample. The non-monogenic samples are labelled as “nonmono” on the y-axes. Supplementary Fig.3 Single-cell RNA sequencing of PBMCs from selected NDD cases versus control: bulk cell analysis of top 5 ORA GO pathways. The top 5 upregulated (red) and t [file mmc1.pdf]

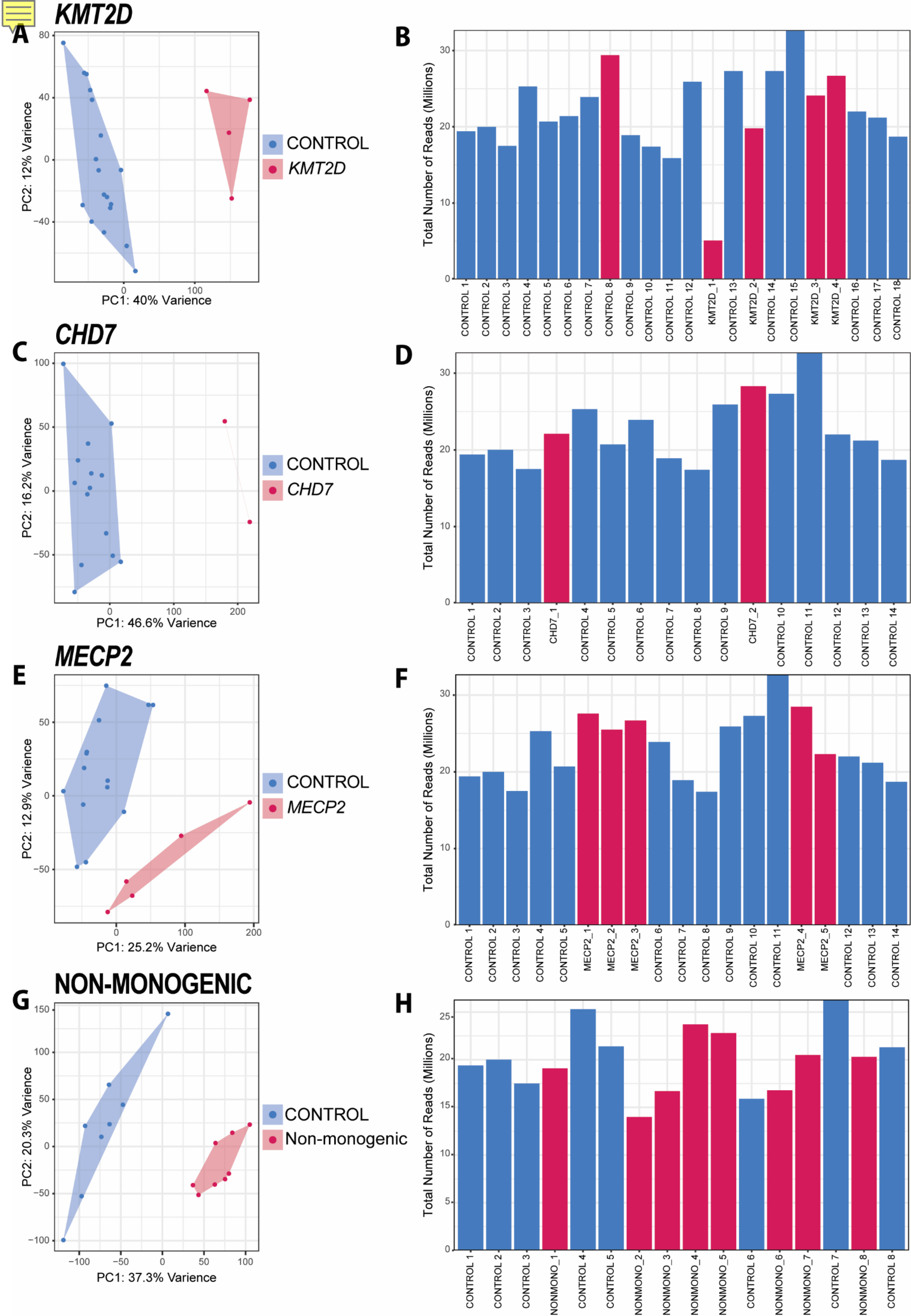
A
KMT2D
PC2: 12% Varience
PC1: 40% Varience
CONTROL
KMT2D
B
Total Number of Reads (Millions)
CONTROL 1
CONTROL 2
CONTROL 3
CONTROL 4
CONTROL 5
CONTROL 6
CONTROL 7
CONTROL 8
CONTROL 9
CONTROL 10
CONTROL 11
CONTROL 12
KMT2D_1
CONTROL 13
KMT2D_2
CONTROL 14
CONTROL 15
KMT2D_3
KMT2D_4
CONTROL 16
CONTROL 17
CONTROL 18
C
CHD7
PC2: 16.2% Varience
PC1: 46.6% Varience
CONTROL
CHD7
D
Total Number of Reads (Millions)
CONTROL 1
CONTROL 2
CONTROL 3
CHD7_1
CONTROL 4
CONTROL 5
CONTROL 6
CONTROL 7
CONTROL 8
CONTROL 9
CHD7_2
CONTROL 10
CONTROL 11
CONTROL 12
CONTROL 13
CONTROL 14
E
MECP2
PC2: 12.9% Varience
PC1: 25.2% Varience
CONTROL
MECP2
F
Total Number of Reads (Millions)
CONTROL 1
CONTROL 2
CONTROL 3
CONTROL 4
CONTROL 5
MECP2_1
MECP2_2
MECP2_3
CONTROL 6
CONTROL 7
CONTROL 8
CONTROL 9
CONTROL 10
CONTROL 11
MECP2_4
MECP2_5
CONTROL 12
CONTROL 13
CONTROL 14
G
NON-MONOGENIC
PC2: 20.3% Varience
PC1: 37.3% Varience
CONTROL
Non-monogenic
H
Total Number of Reads (Millions)
CONTROL 1
CONTROL 2
CONTROL 3
NONMONO_1
CONTROL 4
CONTROL 5
NONMONO_2
NONMONO_3
NONMONO_4
NONMONO_5
CONTROL 6
NONMONO_6
NONMONO_7
CONTROL 7
NONMONO_8
CONTROL 8

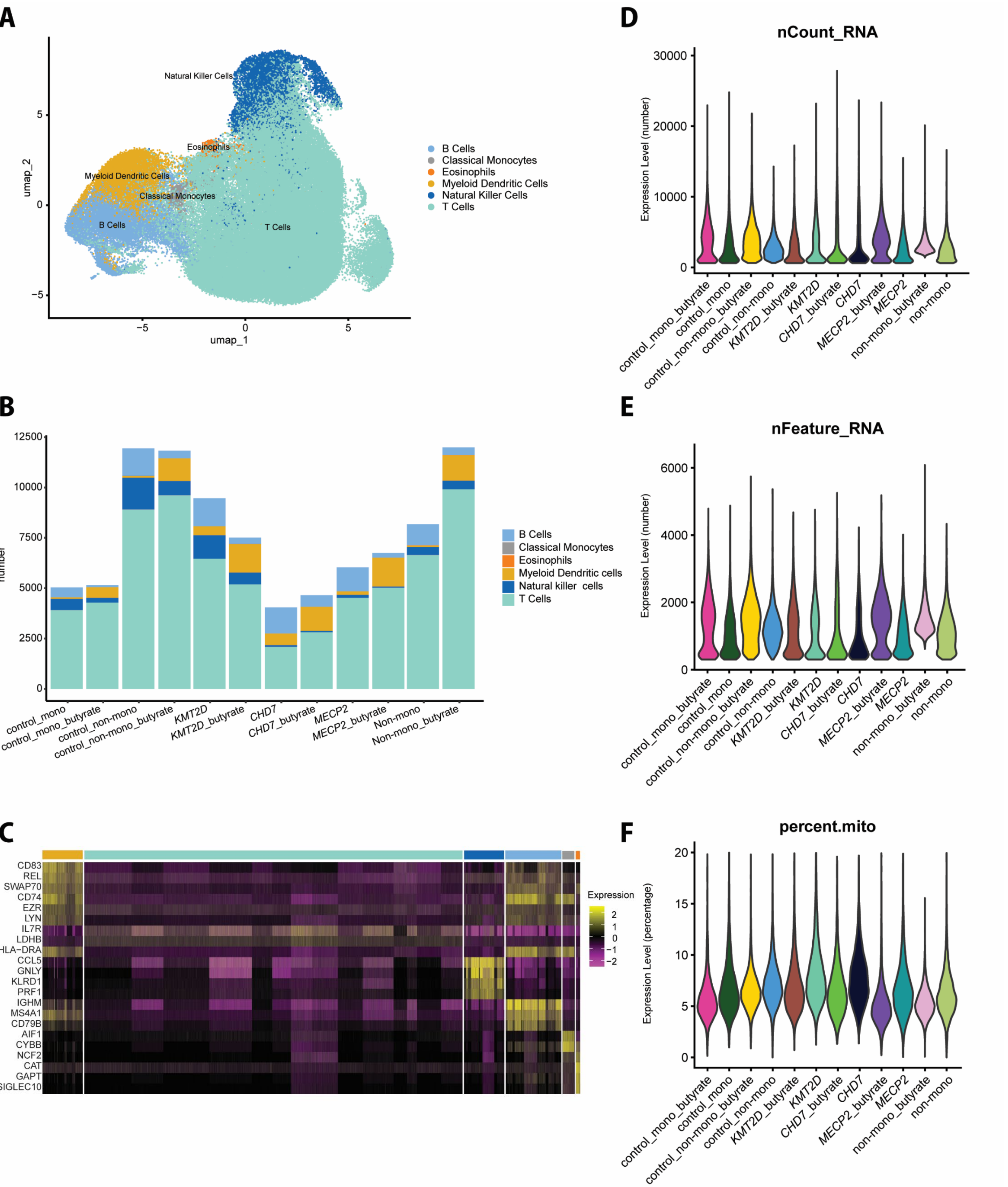
A
Natural Killer Cells
Eosinophils
Myeloid Dendritic Cells
Classical Monocytes
B Cells
T Cells
umap_1
umap_2
B Cells
Classical Monocytes
Eosinophils
Myeloid Dendritic Cells
Natural Killer Cells
T Cells
B
number
control_mono
control_mono_butyrate
control_non-mono
control_non-mono_butyrate
KMT2D
KMT2D_butyrate
CHD7
CHD7_butyrate
MECP2
MECP2_butyrate
Non-mono
Non-mono_butyrate
B Cells
Classical Monocytes
Eosinophils
Myeloid Dendritic cells
Natural killer cells
T Cells
C
CD83
REL
SWAP70
CD74
EZR
LYN
IL7R
LDHB
HLA-DRA
CCL5
GNLY
KLRD1
PRF1
IGHM
MS4A1
CD79B
AIF1
CYBB
NCF2
CAT
GAPT
SIGLEC10
Expression
D
nCount_RNA
Expression Level (number)
E
nFeature_RNA
Expression Level (number)
F
percent.mito
Expression Level (percentage)
control_mono_butyrate
control_mono
control_non-mono_butyrate
control_non-mono
KMT2D_butyrate
KMT2D
CHD7_butyrate
CHD7
MECP2_butyrate
MECP2
non-mono_butyrate
non-mono

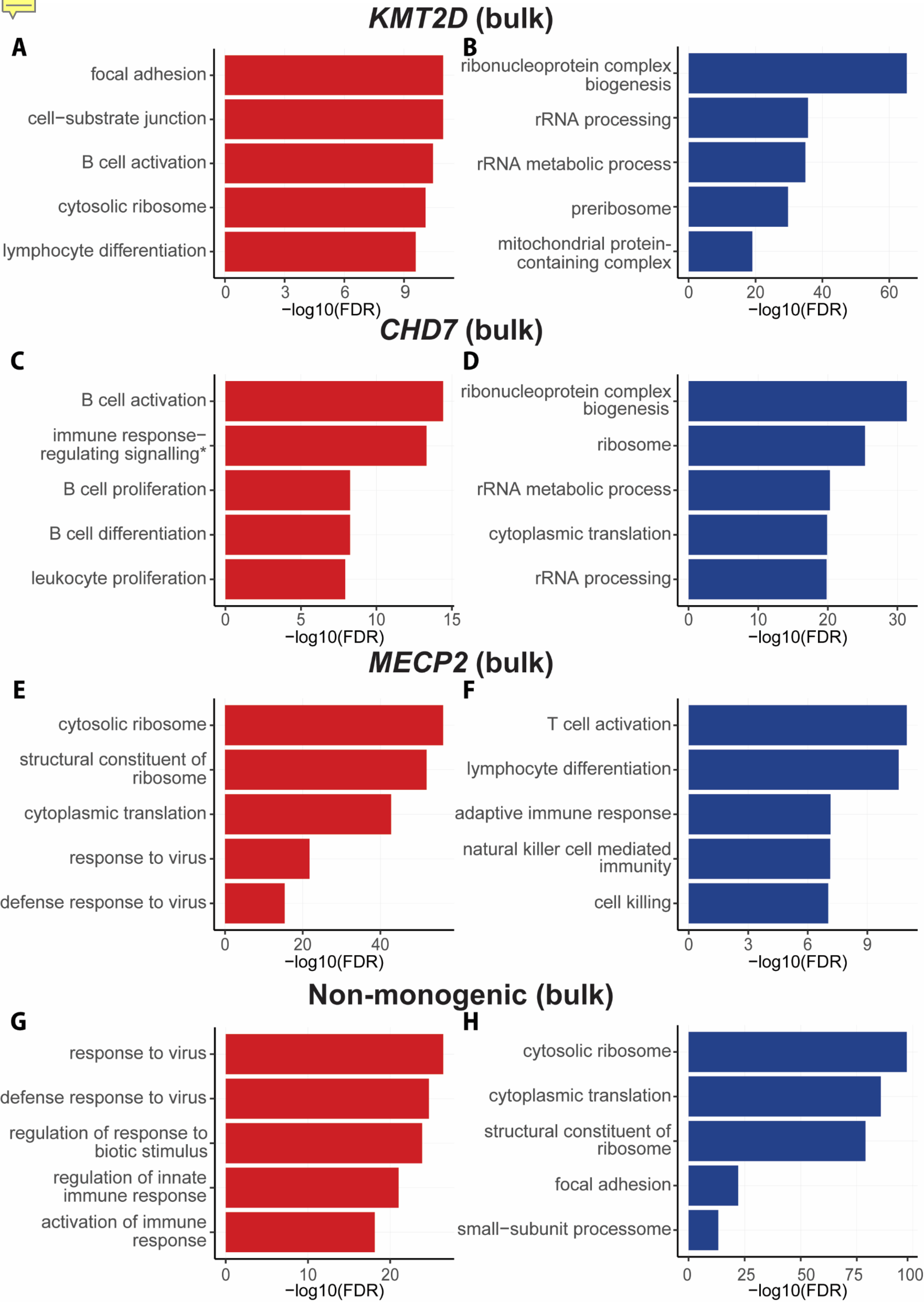
KMT2D (bulk)
A
focal adhesion
cell−substrate junction
B cell activation
cytosolic ribosome
lymphocyte differentiation
0
3
6
9
−log10(FDR)
B
ribonucleoprotein complex biogenesis
rRNA processing
rRNA metabolic process
preribosome
mitochondrial protein-containing complex
0
20
40
60
−log10(FDR)
CHD7 (bulk)
C
B cell activation
immune response−regulating signalling*
B cell proliferation
B cell differentiation
leukocyte proliferation
0
5
10
15
−log10(FDR)
D
ribonucleoprotein complex biogenesis
ribosome
rRNA metabolic process
cytoplasmic translation
rRNA processing
0
10
20
30
−log10(FDR)
MECP2 (bulk)
E
cytosolic ribosome
structural constituent of ribosome
cytoplasmic translation
response to virus
defense response to virus
0
20
40
−log10(FDR)
F
T cell activation
lymphocyte differentiation
adaptive immune response
natural killer cell mediated immunity
cell killing
0
3
6
9
−log10(FDR)
Non-monogenic (bulk)
G
response to virus
defense response to virus
regulation of response to biotic stimulus
regulation of innate immune response
activation of immune response
0
10
20
−log10(FDR)
H
cytosolic ribosome
cytoplasmic translation
structural constituent of ribosome
focal adhesion
small−subunit processome
0
25
50
75
100
−log10(FDR)

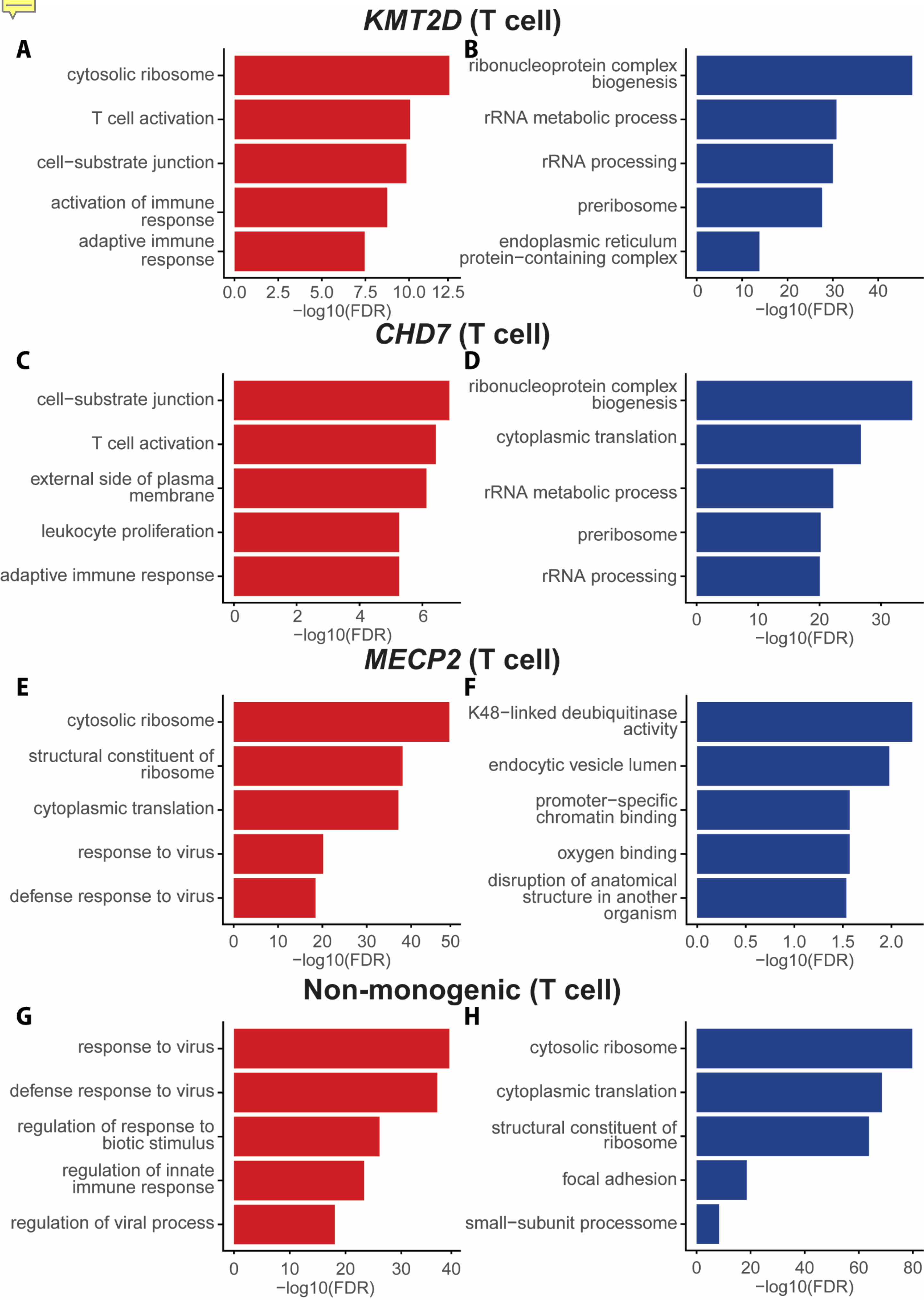
KMT2D (T cell)
A
cytosolic ribosome
T cell activation
cell−substrate junction
activation of immune response
adaptive immune response
0.0 2.5 5.0 7.5 10.0 12.5
−log10(FDR)
B
ribonucleoprotein complex biogenesis
rRNA metabolic process
rRNA processing
preribosome
endoplasmic reticulum protein−containing complex
0 10 20 30 40
−log10(FDR)
CHD7 (T cell)
C
cell−substrate junction
T cell activation
external side of plasma membrane
leukocyte proliferation
adaptive immune response
0 2 4 6
−log10(FDR)
D
ribonucleoprotein complex biogenesis
cytoplasmic translation
rRNA metabolic process
preribosome
rRNA processing
0 10 20 30
−log10(FDR)
MECP2 (T cell)
E
cytosolic ribosome
structural constituent of ribosome
cytoplasmic translation
response to virus
defense response to virus
0 10 20 30 40 50
−log10(FDR)
F
K48−linked deubiquitinase activity
endocytic vesicle lumen
promoter−specific chromatin binding
oxygen binding
disruption of anatomical structure in another organism
0.0 0.5 1.0 1.5 2.0
−log10(FDR)
Non-monogenic (T cell)
G
response to virus
defense response to virus
regulation of response to biotic stimulus
regulation of innate immune response
regulation of viral process
0 10 20 30 40
−log10(FDR)
H
cytosolic ribosome
cytoplasmic translation
structural constituent of ribosome
focal adhesion
small−subunit processome
0 20 40 60 80
−log10(FDR)

Butyrate-treated control versus untreated control

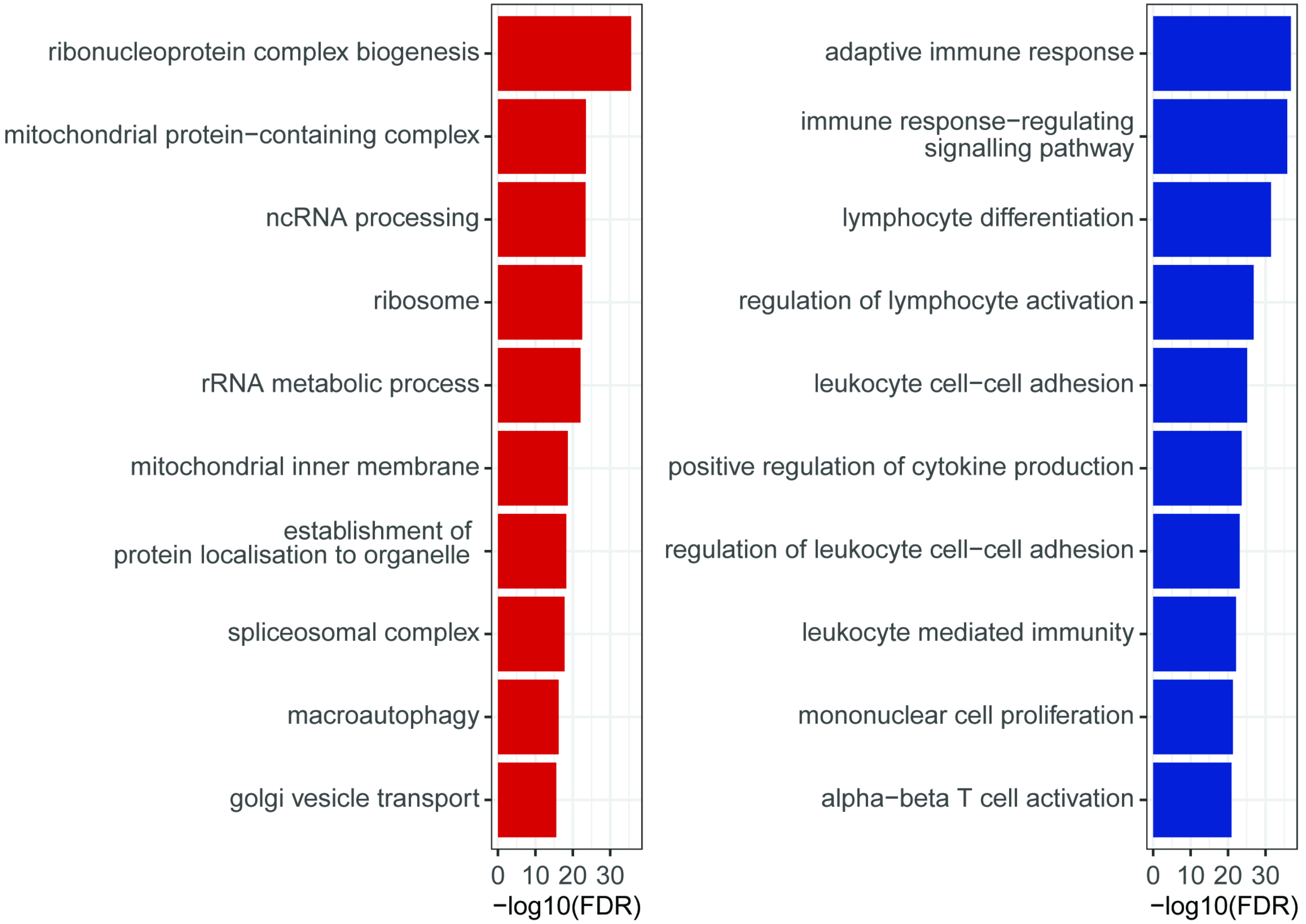

Supplementary Table 1. The number of up- and down-regulated differentially expressed genes (DEGs) of untreated and butyrate-treated PBMCs (bulk) and T cells from NDD patients and controls.

	**BULK**		**T CELL**	
**Comparison**	**Up**	**Down**	**Up**	**Down**
Non-monogenic v control[1]	1365	396	521	402
Non-monogenic_butyrate v non-monogenic	3868	3321	2229	1179
CHD7 v control[2]	1572	2532	821	964
CHD7_butyrate v CHD7	3189	2437	2048	446
KMT2D v control[2]	2044	2726	1794	1699
KMT2D_butyrate v KMT2D	5340	2605	2005	656
MECP2 v control[2]	651	592	323	175
MECP2_butyrate v MECP2	4717	1929	1925	544
Control[2]_butyrate v control[2]	6909	1614	3123	575

Controls for non-monogenic and monogenic NDDs are different and are distinguished as control[1] (for comparison with non-monogenic NDD) and control[2] (for comparison with monogenic NDD).
